# A Critical Review of Kaempferol in Intestinal Health and Diseases

**DOI:** 10.3390/antiox12081642

**Published:** 2023-08-20

**Authors:** Jun Chen, Haopeng Zhong, Zhouyin Huang, Xingping Chen, Jinming You, Tiande Zou

**Affiliations:** Jiangxi Province Key Laboratory of Animal Nutrition, College of Animal Science and Technology, Jiangxi Agricultural University, Nanchang 330045, China; junchen@jxau.edu.cn (J.C.); zhonghaopeng@stu.jxau.edu.cn (H.Z.); hzy11373x@stu.jxau.edu.cn (Z.H.); cxp0315@jxau.edu.cn (X.C.); youjinm@jxau.edu.cn (J.Y.)

**Keywords:** antioxidant, intestinal barrier function, intestinal health, intestinal inflammation, kaempferol

## Abstract

Kaempferol, a secondary metabolite found in plants, is a naturally occurring flavonoid displaying significant potential in various biological activities. The chemical structure of kaempferol is distinguished by the presence of phenyl rings and four hydroxyl substituents, which make it an exceptional radical scavenger. Most recently, an increasing number of studies have demonstrated the significance of kaempferol in the regulation of intestinal function and the mitigation of intestinal inflammation. The focus of the review will primarily be on its impact in terms of antioxidant properties, inflammation, maintenance of intestinal barrier function, and its potential in the treatment of colorectal cancer and obesity. Future research endeavors should additionally give priority to investigating the specific dosage and duration of kaempferol administration for different pathological conditions, while simultaneously conducting deeper investigations into the comprehensible mechanisms of action related to the regulation of aryl hydrocarbon receptor (AhR). This review intends to present novel evidence supporting the utilization of kaempferol in the regulation of gut health and the management of associated diseases.

## 1. Introduction

The gastrointestinal tract represents the most extensive and delicate interface with the external environment [1]. This intricate system simultaneously requires accessibility and permeability to vital nutrients while also safeguarding against harmful pathogens and potentially damaging substances [2]. Most importantly, the intestinal tract constitutes the most extensive immune organ within the human body [3]. However, a growing number of gastrointestinal diseases are afflicting individuals worldwide. In the realm of gastrointestinal illnesses, inflammatory bowel diseases, including Crohn’s disease and ulcerative colitis, represent prevalent intestinal disorders. These conditions pose a potential threat to the overall well-being of the global population [4]. Moreover, colorectal cancer has become a global health concern as its prevalence and mortality rates have been on the rise in recent years. It is anticipated that the worldwide incidence of colorectal cancer will escalate by 60%, resulting in the occurrence of over 2.2 million new cases and causing 1.1 million deaths by the year 2030 [5]. Significantly, an increasing number of studies provide evidence establishing a strong correlation between the functioning of the intestine and the onset and progression of several diseases, such as obesity and arthritis [6,7,8,9].

It has been reported that nutritional or natural antioxidant medical interventions have demonstrated efficacy in enhancing intestinal health and the prevention or treatment of intestinal diseases [10,11]. Flavonoids are a class of polyphenolic compounds frequently present in plant sources, and they represent a substantial component of the human diet [12,13]. Of note, the attention of researchers has been drawn to kaempferol, a plant flavonoid, owing to its diverse bioactive functions. Kaempferol, a naturally occurring flavonol, is extensively present in various vegetables, fruits, and herbal medicines [14]. Kaempferol exhibits various bioactive functionalities, encompassing antioxidant, anti-inflammatory, anti-apoptotic, and anticancer properties [15]. Most recently, a growing body of research has presented compelling evidence regarding the importance of kaempferol in the modulation of intestinal function and in ameliorating intestinal inflammation. Herein, the primary emphasis of the review will be on the potential effects of the antioxidant function, intestinal inflammation, maintenance of intestinal barrier function, and potential in the treatment of colorectal cancer. The objective of this review is to present innovative evidence that supports the usage of kaempferol in the regulation of intestinal functioning and the control of diseases associated with it, such as inflammatory bowel diseases, colorectal cancer, and obesity.

## 2. An Overview of Kaempferol

### 2.1. Physico-Chemical Properties of Kaempferol

**Figure 1:**
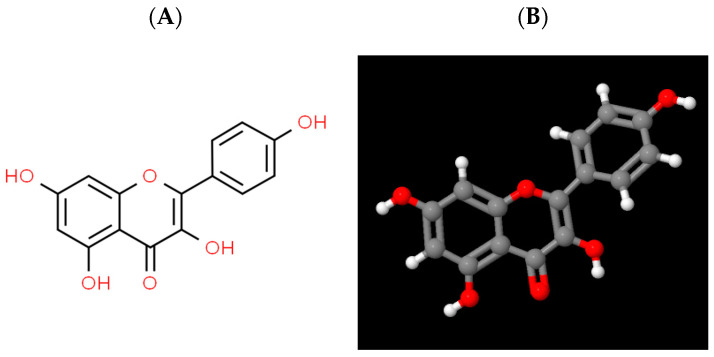
Chemical structure of kaempferol. (**A**). Two-dimensional structure; (**B**). Three-dimensional structure (Source from ChemSpider).

Kaempferol (3,5,7-trihydroxy-2-(4-hydroxyphenyl)-4*H*-chromen-4-one), also known as indigo yellow, is a flavonoid compound with a molecular structural formula of C_15_H_10_O_6_ and a relative molecular weight of 286.23. Its pure product form is a yellow crystalline powder with a melting point of 276–278 °C and an acidity coefficient of 6.34 ± 0.40 [16,17]. It is slightly soluble in water and soluble in organic solvents such as hot ethanol, diethyl ether, and dimethyl sulfoxide. It can be observed that kaempferol possesses a diphenylpropane structure, which is accountable for its hydrophobic characteristic (Figure 1) [18].

### 2.2. Sources of Kaempferol

Kaempferol initially originated from the rhizomes of *Kaempferia galanga* [16]. Furthermore, kaempferol is widely present in various vegetables such as onions, cabbages, and broccoli, as well as in fruits such as strawberries, gooseberries, and blackberries. Additionally, it can be found in herbal medicines such as lovage and barbarum [14,16]. The primary sources of kaempferol in plants that possess the greatest quantity are leafy green vegetables, specifically spinach with a concentration of 55 mg per 100 g, cabbage with 47 mg per 100 g, and broccoli with 7.2 mg per 100 g [19]. Additionally, onions contain 4.5 mg per 100 g, and blueberries have 3.17 mg per 100 g [19]. Black tea is the predominant beverage that contains kaempferol, with 1.7 mg per 100 mL, followed by red wine with 0.23 mg per 100 mL [19]. Spices such as capers contain a notable quantity at 104.29 mg per 100 g, while cumin has 38.6 mg per 100 g, and cloves contain 23.8 mg per 100 g [19]. The extensive dispersion of kaempferol guarantees its accessibility and further implies the potential significance of kaempferol in the dietary habits of individuals.

### 2.3. Absorption and Metabolism of Kaempferol

Kaempferol possesses a relatively high affinity for lipids, and as a result, it is predominantly absorbed in the small intestine via active transport, passive diffusion, and facilitated diffusion [16,20]. According to one report, the concentration of kaempferol excreted in the urine was found to be 2.5% of the amount consumed by the recruited participants [21]. Another study administered a dose of 12.5 mg of kaempferol for 12 days and observed a rate of 0.9% kaempferol excretion in the urine [22]. In addition, the consumption of cooked endive (a leafy vegetable categorized under the genus *Cichorium*) resulted in a urinary excretion of 1.9% within 24 h when taking 9 mg of kaempferol. Furthermore, the peak concentration in plasma reached 0.1 μM after 5.8 h [23].

Research conducted on the metabolism and excretion of kaempferol has indicated that it undergoes primary metabolism within the liver and subsequently circulates in the bloodstream in the form of methyl, sulfate, or glucuronide conjugates [24,25]. In the interim, intestinal microbiota possess the ability to metabolize kaempferol glycosides primarily into aglycones. This metabolic process further encompasses the conversion of these aglycones into three substances, namely 4-hydroxyphenylacetic acid, 4-methylphenol, and phloroglucinol. After this metabolic transformation, these substances are absorbed into systemic circulation, after which they are distributed throughout various tissues. Eventually, these substances are eliminated from the body via either feces or urine [21,22]. Furthermore, it has been discovered that kaempferol possesses the ability to undergo metabolism, resulting in the production of ubiquinone within kidney cells [26]. Even though it has been reported that kaempferol-3-glucuronide is the primary metabolite found in plasma and urine, the presence of kaempferol monosulfate and disulfates has also been observed in urine [23]. Additionally, the concentration of unbound kaempferol in urine was found to be notably lower than in blood, suggesting that a portion of the aglycon is metabolized within the kidney before being eliminated.

### 2.4. Biological Functions of Kaempferol

**Figure 2:**
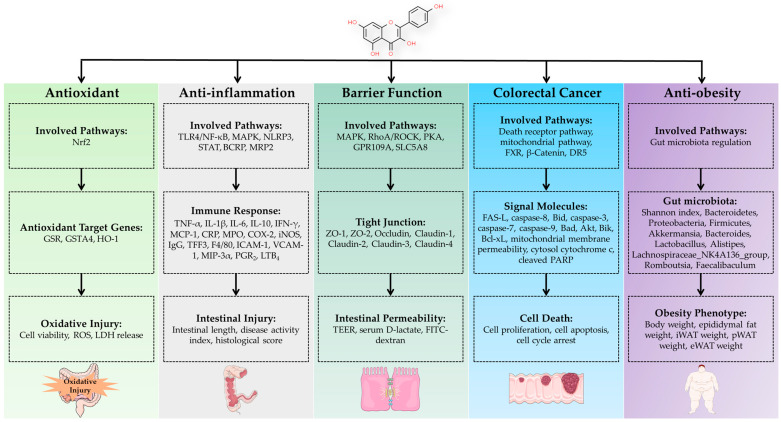
The beneficial effects of kaempferol on the intestine and the proposed mechanism of action based on current knowledge. Abbreviations: BCRP, breast cancer resistance protein; COX-2, cyclooxygenase-2; CRP, C-reaction protein; DR5, death receptor 5; eWAT, epididymal white adipose tissue; FXR, farnesoid X receptor; GSR, glutathione reductase; GSTA4, glutathione S-transferase 4; HO-1, heme oxygenase-1; ICAM-1, intercellular adhesion molecule-1; IFN-γ, interferon-γ; IL-1β, interleukin-1β; IL-6, interleukin-6; IL-10, interleukin-10; iONS, inducible nitric oxide synthase; iWAT, inguinal white adipose tissue; LDH, lactate dehydrogenase; LTB_4_, leukotriene B_4_; MAPK, mitogen-activated protein kinase; MCP-1, monocyte chemoattractant protein-1; MIP-3α, macrophage inflammatory protein-3 alpha; MPO, myeloperoxidase; MRP2, multi-drug resistance-associated protein 2; NF-κB, nuclear factor kappa-B; NLRP3, nucleotide oligomerization domain (NOD)-like receptor 3; Nrf2, nuclear factor-E2-related factor 2; PARP, poly ADPribose polymerase; PGE_2_, prostaglandin E_2_; PKA, protein kinase A; pWAT, perirenal white adipose tissue; RhoA/ROCK, Ras homolog gene family member A/Rho-associated protein kinase; ROS, reactive oxygen species; STAT, signal transducer and activator of transcription; TEER, transepithelial electrical resistance; TFF3, trefoil factor family 3; TLR4, toll-like receptor 4; TNF-α, tumor necrosis factor-α; VCAM-1, vascular cell adhesion molecule-1.

There has been substantial research conducted on kaempferol due to its various bioactivities, such as its antioxidant, antimicrobial, anti-inflammatory, and anticancer properties [27]. Kaempferol, being a representative flavonoid, shares some inherent attributes typical of flavonoids [27]. Flavonoids are naturally occurring polyphenolic compounds that have been demonstrated to possess a range of favorable health effects, mainly attributable to their antioxidative properties [27]. The protective impact of flavonoids arises from their capacity to initially undergo oxidation and thus convert free radicals into stable, deprotonated forms [27]. The antioxidative activity of flavonoids is of considerable significance due to several factors. By harnessing their antioxidative properties, flavonoids can fulfill several functions, including binding with metal ions, disrupting enzymes involved in the production of free radicals, and directly neutralizing free radicals as antioxidants. Depending on their bioavailability and chemical attributes, they can counteract various oxygen species generated during oxidative stress [27]. Interestingly, kaempferol possesses distinctive regulatory functions in the regulation of intestinal health and the management of intestinal diseases. The following section, serving as the main body of this review, will present a systematic overview of the most recent progress in kaempferol concerning intestinal health and disease, with a primary emphasis on its pivotal function in enhancing the intestinal antioxidant capacity, alleviating gut inflammation, fortifying the integrity of the intestinal barrier, and combatting colorectal cancer. Figure 2 summarizes the beneficial effects of kaempferol on the intestine and the proposed mechanism of action based on current knowledge.

## 3. Regulation of Intestinal Health by Kaempferol

### 3.1. Regulation of Intestinal Antioxidant Function

Oxidative stress is a state of imbalance between cellular oxidants and antioxidants, leading to inadequate elimination of reactive oxygen (ROS) and nitrogen species (RNS) produced within the cells [28]. The excessive production of ROS and RNS within cells leads to the occurrence of oxidative stress, thereby causing damage to cellular proteins, lipids, and DNA [29]. Kaempferol, a commonly used flavonoid, exhibits high antioxidant activity due to the abundance of phenolic hydroxyl groups in its structure [30]. Several studies have provided evidence of kaempferol’s antioxidant activity by demonstrating its ability to scavenge radicals in oxygen-damaged erythrocytes, leading to the generation of reactive oxygen species [27].

In a study, it was revealed that treating IEC-6 cells (rat intestinal epithelial cells) with 5 μmol/L of kaempferol for 24 h resulted in a significant reduction in oxidative damage and cell cytotoxicity when challenged with indomethacin [31]. More specifically, the kaempferol treatment led to a decrease in cellular ROS production, lactate dehydrogenase (LDH) release, and Ca^2+^ levels while increasing cell viability [31]. Similarly, another study revealed the potential of a 10 μmol/L kaempferol treatment for 24 h in ameliorating oxidative damage and apoptosis in IPEC-1 cells (intestinal porcine epithelial cells) that were challenged with diquat [32]. The treatment with kaempferol resulted in a decrease in cellular ROS production and *GCLC* mRNA levels while simultaneously increasing T-Nrf2 protein levels as well as *GSR*, *GSTA4*, and *HO-1* mRNA levels [32]. Furthermore, the kaempferol treatment produced a decrease in cell apoptosis, mitochondrial depolarization, and p-JNK/JNK protein levels, along with an increase in Bcl-2/Bax and PARP-1 protein levels in the cells [32]. Additionally, the treatment with kaempferol led to an increase in cell viability and cell migration, along with a decrease in G1 phase arrest and an increase in G2/M phase arrest, as well as *Cyclin D1*, *CDK4*, *CDK6* mRNA levels, and β-catenin protein level in the cells [32].

Based on the aforementioned results, kaempferol has the potential to augment the antioxidant capacity and reduce oxidative stress in intestinal epithelial cells. These positive effects can be mainly attributed to its activation of the Nrf2 pathway, which is considered the primary cellular endogenous pathway for antioxidants [33]. Once the Nrf2 pathway is activated by kaempferol, the transcription of downstream target genes related to antioxidants is enhanced, thereby promoting intestinal antioxidant functionality. Nevertheless, it is important to note that these validation results have been obtained solely from in vitro studies, and thus further investigations are required in vivo to authenticate the potential antioxidant properties in the intestine.

### 3.2. Regulation of Intestinal Inflammation

Inflammation is an essential biological response that is accompanied by symptoms including pain, redness, and heat. This response frequently leads to the typical physiological impairment of the affected tissues [34], such as inflammatory bowel diseases encompassing Crohn’s disease and ulcerative colitis [33]. Numerous studies have documented the anti-inflammatory properties of kaempferol, both in in vivo and in vitro studies.

One approach for drug-giving is oral administration, and the ingestion of kaempferol as a means of oral administration has been documented as a viable technique for alleviating intestinal inflammation. In a recent study, it was observed that the oral administration of kaempferol at a dose of 100 mg/kg effectively inhibited acute colitis and extended the length of the colon in mice treated with dextran sulfate sodium (DSS). The authors provided evidence supporting the proposition that the positive impacts of kaempferol on colitis are mediated via the regulation of the efflux transporters BCRP (breast cancer resistance protein) and MRP2 (multi-drug resistance associated protein 2) [35]. Most recently, it was found that the oral administration of kaempferol at doses of 25, 50, or 100 mg/kg/day resulted in a reduction of inflammation in the colon of C57BL/6J mice with chronic colitis induced by DSS. This reduction was evident from a decrease in the levels of colonic IL-1β, IL-6, and TNF-α, as well as colonic CRP (C-reaction protein) and MPO (myeloperoxidase) activity [36]. Notably, the administration of kaempferol also led to a decrease in disease activity index and the colonic H&E score of the mice [36]. Consistently, it was also documented that oral administration of kaempferol at a dosage of 50 mg/kg/day resulted in decreased levels of IL-1β, IL-6, and TNF-α in the serum of DSS-challenged mice. Additionally, there was a decrease in the mRNA levels of *IL-1β*, *IL-6*, *TNF-α*, *COX-2*, *MCP-1*, *iNOS*, *TLR4*, *NLRP3*, *MAPK*, and *NF-κB*, while there was an increase in the mRNA level of *IL-10* in the colonic tissues of mice with colitis induced by DSS [37]. Furthermore, the activation of the LPS-TLR4-NF-κB pathway was observed, as indicated by the reduction in serum LPS level and the protein levels of TLR4, MyD88, p-NF-κB-P65, and NLRP3 in colon tissues [37]. Significantly, the administration of kaempferol resulted in an augmentation of colon length and a reduction in disease activity index and colon histological score of mice afflicted with colitis induced by DSS [37]. More interestingly, another study discovered that gavage administration of kaempferol at a dosage of 200 mg/kg/day led to a reduction in inflammation in mice with collagen-induced arthritis. This reduction was observed in various markers such as plasma IL-1β, TNF-α, IL-6, IFN-γ, and IgG levels [8]. Additionally, the authors noted that the body weight of the mice increased, while parameters such as paw thickness, polyarthritis index, and spleen index decreased [8].

The dietary supplementation of kaempferol has also been found to ameliorate intestinal inflammation induced by DSS and obesity in a mouse model [38,39]. It was reported that when mice were given a dietary supplement containing 0.3% kaempferol for 3 weeks, there was a reduction in the inflammation response induced by the DSS challenge in mice. This was indicated by decreased levels of plasma PGE_2_ (prostaglandin E_2_) and LTB_4_ (leukotriene B_4_), as well as a decrease in colonic mucosa MPO level. The study also found that the mRNA levels of *COX-2*, *iNOS*, *TNF-α*, *IL-1β*, and *IL-6* were downregulated, whereas the mRNA level of *TFF3* was upregulated in the colonic mucosa of mice receiving the 0.3% kaempferol supplementation [38]. Regarding phenotype, kaempferol supplementation resulted in an increase in colonic length as well as a decrease in disease activity index, colonic histological score, and spleen weight of mice [38]. In an experimental model of obesity induced by a high-fat diet, the addition of 0.1% kaempferol to the diet over a period of 16 weeks resulted in a reduction of intestinal inflammation in mice. This was evident from a decrease in the levels of macrophage cells, dendritic cells, MPO, and *F4/80*, *IL-6*, *TNF-α*, *MCP-1*, and *IL-1β* mRNA levels in the colon tissue of the mice [39]. The supplementation with kaempferol was found to activate the TLR4/NF-κB pathway, as indicated by decreased protein levels of TLR4, MyD88, cytosolic p65, and nuclear p65 in the colon tissue of the mice [39]. In terms of phenotype, the inclusion of kaempferol in the diet led to a reduction in body weight and epididymal fat weight in mice that were given a high-fat diet [39].

The anti-inflammatory properties of kaempferol on the intestine were further demonstrated in in vitro investigations. According to a recent study, the administration of 50 μmol/L kaempferol for a duration of 3 h significantly reduced the production of cell cytokines (TNF-α, IL-1β, IL-6 levels in the culture supernatant) and adhesion proteins (ICAM-1, VCAM-1 level in the culture supernatant) in rat intestinal microvascular endothelial cells that were subjected to challenge by LPS (lipopolysaccharide) [40]. As for the mechanism of action, the administration of kaempferol inhibited several pathways, such as the decreased protein levels of TLR4, p-NF-κB p65/NF-κB p65, p-I-κB/I-κB, p-STAT/STAT in the cells [40]. In another study, it was found that treating rat intestinal microvascular endothelial cells with a concentration of 100 μmol/L kaempferol for 24 h resulted in a reduction of cell inflammation when exposed to LPS and TNF-α co-challenge [41]. This treatment led to decreased levels of IL-6 in the culture supernatant, as well as down-regulation of the protein level of p-p65/p65, and the mRNA levels of *IL-6* and *RELA/p65*. The treatment also led to an increase in the levels of IL-10 in the culture supernatant [41]. More interestingly, according to the findings of a previous study, the exposure of Caco-2 cells to kaempferol at a concentration of 20 μmol/L for a duration of 24 h resulted in the suppression of immunoglobulin E-mediated allergic inflammation. This suppression was evidenced by a reduction in levels of IL-8 and MIP-3α (macrophage inflammatory protein-3 alpha), as well as a down-regulation of the p-ERK protein level [42].

Based on the in vivo and in vitro studies mentioned above, it is evident that kaempferol possesses the potential to alleviate intestinal inflammation across various inflammation models, such as LPS, TNF-α, DSS, and high-fat diet-induced inflammation. Nevertheless, further clinical studies are imperative to endorse the implementation of kaempferol as a preventive or therapeutic agent for intestinal ailments associated with inflammation.

### 3.3. Regulation of Intestinal Barrier function

The intestine serves as the primary organ responsible for the absorption of nutrients and water, making it the most extensive region in contact with environmental elements [43]. The intestinal mucosal surface is composed of epithelial cells, which function as a highly efficient barrier through intercellular junctions, segregating the inner and outer environments while preventing the entry of potentially dangerous substances [44]. The association between the impairment of intestinal barrier function and common diseases, particularly inflammatory bowel diseases, has been significantly demonstrated [45]. There is a growing body of evidence suggesting that kaempferol has the potential to improve intestinal barrier function, as indicated by both in vivo and in vitro studies.

It was discovered that the oral administration of doses of 25, 50, or 100 mg/kg/day effectively reduced the levels of serum D-lactate and FITC-dextran in C57BL/6J mice with chronic colitis induced by DSS [36]. This decrease signifies an enhancement in the barrier function. Consistently, the administration of a 0.1% kaempferol dietary supplement led to improvements in intestinal barrier integrity [39]. These enhancements were indicated by reduced levels of FITC-dextran in the serum, as well as increased mRNA expression of ZO-1, Occludin, and Claudin-1 within the colon tissue of mice subjected to the kaempferol supplementation [39]. Furthermore, in a recent study, it was observed that the oral administration of kaempferol at a dosage of 50 mg/kg/day contributed to the enhancement of the intestinal barrier integrity of mice. This improvement was evidenced by a reduction in serum FITC-dextran levels, as well as an increase in the mRNA and protein levels of ZO-1, Occludin, and Claudin-1 in colonic tissues [37]. It was found that the oral administration of either 50 or 100 mg/kg of kaempferol resulted in elevated protein expression levels of Occludin and ZO-1 in the ileum and colon of mice suffering from acute liver injury caused by alcohol [46]. The administration of kaempferol also led to an increase in the protein expression levels of the butyrate receptor (GPR109A) and transporter (SLC5A8) in the ileum and colon of the mice [46].

In vitro studies further confirm the advantageous impact of kaempferol on the intestinal barrier function. Suzuki and colleagues (2011) conducted a study in which they observed that the treatment of Caco-2 cells with 100 μmol/L kaempferol for a duration of 48 h resulted in an improvement in intestinal barrier function [47]. The researchers discovered that this treatment led to an increase in the levels of TEER (transepithelial electrical resistance), ZO-2, and Claudin-4 protein levels in the entire cell lysate [47]. Additionally, the kaempferol treatment also increased the protein levels of ZO-1, ZO-2, Occludin, Claudin-1, Claudin-3, and Claudin-4 in the cell detergent-insoluble fraction [47]. Similarly, it was observed that treatment with a concentration of 5 μmol/L of kaempferol for a duration of 24 h resulted in an increase in epithelial barrier function [48]. This increase manifested in elevated levels of ZO-1, Occludin, and Claudin-1 mRNA and protein in the treated cells. The researchers also discovered that the kaempferol treatment enhanced antibacterial activity and reduced the translocation of *E. coli* in IEC-6 cells [48]. The authors further demonstrated that the positive effects of kaempferol are mediated via the RhoA/ROCK (Ras homolog gene family member A/Rho-associated protein kinase) signaling pathway, which is acknowledged to be involved in cellular barrier function [48]. 

The impairment of the intestinal barrier function has been documented in diquat [32], indomethacin [31], LPS [49], LPS and TNF-α [41], and deoxynivalenol [50,51]-induced models of intestinal cells. It has been reported that kaempferol exhibits the ability to alleviate the intestinal barrier dysfunction caused by these stimuli. In a model involving IPEC-1 cells and induced oxidative stress caused by diquat, the treatment with 10 μmol/L kaempferol for a duration of 24 h resulted in the improvement of epithelial barrier function. This improvement was observed from increased levels of TEER, ZO-1, ZO-2, Occludin, and Claudin-4 protein expression levels, as well as a decrease in FITC-dextran [32]. In a recently published study, it was observed that a 24-h treatment with 5 μmol/L kaempferol resulted in an increase in TEER and a decrease in FD-4 in IEC-6 cells when challenged with indomethacin [31]. The authors discovered that the mRNA and protein levels of ZO-1, Occludin, and Claudin-1 were upregulated as a result of the kaempferol treatment [31]. It was also noted that the positive effects of kaempferol were mediated through the activation of JNK/Src [31]. In a coculture of Caco-2 cells combined with rat intestinal microvascular endothelial cells, the treatment with kaempferol at a concentration of 80 μmol/L for a duration of 48 h resulted in an improvement in barrier function and a reduction in inflammation caused by LPS [49]. The researchers found that kaempferol treatment led to a decrease in FITC and IL-8 levels, as well as an increase in TEER, ZO-1, Occludin, and Claudin-2 protein levels in the cocultured cells. They also observed that kaempferol inhibited the protein levels of p-p65/p65 and p-I-κB/I-κB in the cocultured cells [49]. Interestingly, in a cell model where LPS and TNF-α were simultaneously applied, it was observed that exposing rat intestinal microvascular endothelial cells to a 24-h treatment of kaempferol at a concentration of 100 μmol/L led to heightened TEER, ZO-1, and Occludin protein levels [41]. Additionally, there was a decrease in the permeability of EB-Albumin and Claudin-2 protein levels in cells [41]. In a study using a Caco-2 cells model exposed to deoxynivalenol, it was found that treatment with kaempferol had varying effects on the expression of tight proteins at different stages of cell proliferation and differentiation [50]. During the proliferation stage, kaempferol treatment resulted in a decrease in the level of ZO-1 protein and an increase in FD-4 flux, as well as an increase in Claudin-3 protein level [50]. On the other hand, during the intermediate stage, kaempferol treatment led to an increase in TEER and Claudin-3 protein levels and a decrease in FD-4 flux, as well as a decrease in Claudin-4 protein level [50]. Lastly, during the differentiated stage, kaempferol treatment resulted in an increase in TEER, as well as increases in Claudin-3 and ZO-1 protein levels in Caco-2 cells [50]. Importantly, it was discovered that the treatment of Caco-2 cells with kaempferol at a concentration of 100 μmol/L for a duration of 24 h successfully alleviated the deoxynivalenol-induced dysfunction of the intestinal barrier [51]. Via the utilization of proteomic analysis, the authors identified that the proteins showing differential expression were primarily associated with cell adhesion molecule binding, cell junctions, and the assembly of cell junctions [51]. Further investigation revealed that the treatment with kaempferol influenced the expression and assembly of proteins involved in tight junctions and adherens junctions via the PKA pathway and the MAPK/ERK pathway, thereby enhancing the integrity of the Caco-2 cell monolayer [51].

In this context, kaempferol has the ability to preserve the integrity of the intestinal barrier both in vivo and in vitro. Additionally, it possesses the ability to alleviate disruptions in the intestinal barrier that are induced by various stimuli, such as diquat, indomethacin, LPS, TNF-α, and deoxynivalenol. Nonetheless, the ongoing studies are presently constrained solely to mouse models and in vitro cell models, thus necessitating further human trials to authenticate the impact of introducing kaempferol.

### 3.4. Treatment of Colorectal Cancer

According to multiple research sources, it has been proposed that kaempferol demonstrates promising qualities as an anticancer agent [27]. It was reported that administering 50 mg/kg of kaempferol via gavage over a period of 6 weeks resulted in a reduction in the number of polyps in the intestinal tissue of *Apc^Min/+^ mice* [52]. The administration of kaempferol was observed to decrease the mRNA level of *TNF-α* in the ileum of the mice. Moreover, it was noted that the levels of FXR mRNA and protein were upregulated, while the level of β-Catenin protein in the intestinal tissues was downregulated due to kaempferol administration [52].

Multiple studies have been conducted at the cellular level regarding kaempferol’s impact on colorectal cancer. According to Cho and Park’s research conducted in 2013, it was observed that the administration of kaempferol led to a decline in cell proliferation and an increase in cell cycle arrest in HT-29 cells with the mutant *p53* gene (human colon cancer cells) [53]. In another study, the administration of kaempferol resulted in an elevation in cell apoptosis, coupled with the activation of PARP and caspase-3 cleavage in HCT116 cells (human colon cancer cells) [54]. Moreover, it has been reported that kaempferol sensitizes SW480 cells, specifically human colon cancer cells, to apoptosis induced by TRAIL [55]. The authors noted a significant increase in apoptosis when the combination of kaempferol and TRAIL was used [55]. The researchers also observed that kaempferol specifically upregulated the TRAIL receptors DR4 and DR5. Blocking DR5, but not DR4, using siRNA effectively prevented apoptosis caused by the co-administration of kaempferol and TRAIL [55]. These findings suggest that the upregulation of DR5 by kaempferol contributes to the enhancement of TRAIL’s apoptotic effects [55]. Consistent with these findings, the treatment with 60 μmol/L kaempferol for 48 h resulted in an increase in early apoptotic cells in HT-29 cells [56]. Additionally, the authors observed that the apoptotic-inducing effects of kaempferol are mediated via various pathways. These pathways include the caspase pathway, as evidenced by the increase in protein levels of cleaved PARP, cleaved caspase-3, cleaved caspase-7, and cleaved caspase-9 in cells. The intrinsic apoptotic pathway was also involved, accompanied by decreased protein levels of Bcl-xL and p-Akt, as well as increased protein levels of Bik and the mitochondria Bad. Furthermore, there was a decrease in Akt activity, an increase in mitochondrial membrane permeability, and an increase in cytosol cytochrome c protein levels. Lastly, the extrinsic apoptotic pathway played a role, as indicated by the increased levels of FAS-L, decreased protein levels of caspase-8 and Bid, and increased activity of caspase-8 [56].

In light of the existing evidence, it is believed that kaempferol holds significant promise as a novel agent for the treatment of colorectal cancer. However, further extensive research is required to investigate the clinical trials and underlying mechanisms of action.

### 3.5. Anti-Obesity via Regulation of Gut Microbiota

The link between intestinal microbiota and metabolic disorder diseases, particularly in the case of obesity, has been evidenced in recent years [57]. The data gleaned from both human and animal studies provide empirical evidence pointing towards a strong correlation between the development of an obese phenotype and the gut microbiome [57]. The gut microbiota has enormous metabolic capacity, behaving as a central modulator in contributing to obesity. Most importantly, recent studies have revealed that polyphenols are capable of modulating the composition of the intestinal microbiota and their metabolites, consequently improving metabolic disorders [58]. Of note, the attention of researchers has been drawn to kaempferol owing to its regulatory potential on gut microbiota. The key role of kaempferol was highlighted in preserving the gut microbiota by documenting the effects of kaempferol on diminishing DSS-induced chronic colitis in C57BL/6J mice [36]. Using the fecal microbiota transplantation method, the modulatory effects of kaempferol was confirmed on murine experimental colitis in C57BL/6J mice, which is strongly associated with the modification of the gut microbiota [37]. The aforementioned results indicate that kaempferol possesses the ability to regulate gut microbiota.

Kaempferol is involved in the regulation of obesity via the regulation of the microbiota. In a study, it was discovered that the addition of kaempferol at a dosage of 200 mg/kg in the diet for a duration of 8 weeks led to a reduction in body weight, as well as the weights of inguinal white adipose tissue, epididymal white adipose tissue, and perirenal white adipose tissue in C57BL/6J mice that were subjected to a high-fat diet [59]. The authors further found that kaempferol supplementation resulted in an elevation of the Shannon index in fecal samples from mice fed a high-fat diet, which is indicative of an increased alpha-diversity of gut microbiota due to kaempferol supplementation [59]. Furthermore, supplementation with kaempferol resulted in an augmentation of the relative abundance of *Bacteroidetes* and *Proteobacteria*, as well as a reduction in the relative abundance of *Firmicutes* at the phylum level in the collected fecal samples. Moreover, at the genus level, supplementation with kaempferol led to an increase in the relative abundance of *Akkermansia*, *Bacteroides*, and *Lactobacillus* in the fecal samples of mice that were fed a high-fat diet [59]. In another obese mouse model, dietary supplementation with 0.1% kaempferol for 16 weeks reduced obesity associated with the regulation of gut microbiota in C57BL/6J mice that were fed a high-fat diet [39]. The investigators observed that the addition of kaempferol to the diet resulted in an elevated level of microbial diversity in mice fed a high-fat diet. At the level of the phylum, the administration of kaempferol entirely averted the rise in *Firmicutes* and the decline in *Bacteroidetes* caused by the high-fat diet. The ratio of *Firmicutes* to *Bacteroidetes* (F/B) escalated with the consumption of the high-fat diet. Conversely, the supplementation of kaempferol diminished the F/B ratio to a degree comparable to the level seen in mice from the control group. Kaempferol also effectively counteracted the alterations induced by a high-fat diet in *Rikenallaceae*, *Prevotellaceae*, *Desulfovibrionaceae*, and *Helicobacteraceae* at the family level. At the genus level, the inclusion of kaempferol significantly mitigated the alterations observed in *Alistipes*, *Lachnospiraceae_NK4A136_group*, *Romboutsia*, and *Faecalibaculum*. Moreover, kaempferol substantially reversed the modifications induced by a high-fat diet in *Desulfovibrio*, *Helicobacter*, and *Akkermansia*. The authors employed Spearman’s correlation analysis to demonstrate that the prevalence of various bacterial strains had a significant correlation with physiological indicators linked to obesity. Specifically, *Parasutterella*, *Alloprevotella*, and *Akkermansia* displayed a negative correlation with obesity-associated indices. These findings suggest that these particular strains might exert a crucial influence in mitigating obesity [39].

Hence, kaempferol participates in the regulation of obesity via the regulation of gut microbiota. The anti-obesity property of kaempferol also suggests that the gut microbiota could be regarded as a potential target for tackling obesity, with the aim of investigating potential agents, particularly plant-derived polyphenols, to combat obesity.

### 3.6. Regulation of Other Physiological Functions

Additional physiological functions of kaempferol in the intestine have been documented as well. It was reported that oral administration of 25, 50, or 100 mg/kg/day demonstrated a mitigating effect on intestinal angiogenesis in mice. This was evidenced by a reduction in VEGF-A (vascular endothelial growth factor-A) and NO levels, an increase in IL-22BP (interleukin-22 binding protein) protein level, and a decrease in VEGFR2 (vascular endothelial growth factor receptor 2), COX-2 (cyclooxygenase-2), TRAF6 (TNF receptor-associated factor 6) protein levels, as well as VEGFR2 (IHC score) and microvascular density of the mice [36]. In a recent study, it was observed that the pretreatment of rat intestinal microvascular endothelial cells with 100 μmol/L kaempferol for a duration of 24 h effectively hindered angiogenesis initiated by LPS and TNF-α [41]. The researchers discovered that this pretreatment with kaempferol resulted in decreased levels of *VEGFR-2*, *VEGF* mRNA, VEGFR-2, p-Akt/Akt, VEGF-A, HIF-1α (hypoxia-inducible factor 1α), and p-p38/p38 protein in the cells [41]. Furthermore, pretreatment with kaempferol also prevented the formation of intestinal microvessels, as evidenced by reduced tube nodes and total branching length [41]. Additionally, it was observed that kaempferol pretreatment inhibited cell migration, as supported by decreased cell mobility and reduced protein levels of p-p38 and p-HSP27 [41]. In another study, it was found that oral administration of 50 or 100 mg/kg of kaempferol for a duration of 2 weeks resulted in a significant reduction in histological scoring of the ileum and colon in a model of acute alcoholic liver injury. This reduction was observed in various parameters including severity, degree of edema, and location in the ileum and colon of mice [46]. The administration of kaempferol was also shown to lower the histological scoring of the liver in mice, specifically in relation to interface hepatitis, lobular disarray, lobular inflammation, steatosis, and apoptosis [46].

These recent functional findings expand the range of potential applications of kaempferol while enhancing our comprehension of this compound. They provide compelling evidence for the need to conduct further comprehensive research on kaempferol’s role in regulating intestinal function. The condensation of kaempferol’s effectiveness as a therapeutic agent for intestinal health and diseases demonstrated in both in vivo and in vitro studies, has been compiled and presented in Table 1 and Table 2, respectively.

## 4. Future Research Directions

Regarding the further investigation of the mechanism of action, more attention should be given to the modulatory effects of kaempferol on gut microbiota and microbiota-derived metabolites. The role of gut microbiota in intestinal inflammation and intestinal barrier function, and consequently the overall health of the host body, has been firmly established [39,60,61]. In recent years, there has been a growing body of evidence highlighting the importance of microbial metabolites, such as those produced via tryptophan metabolism and bile acid metabolism. It was reported that the mitigation effect of kaempferol on tumor burden in *Apc^Min^*^/+^ mice is achieved via the modulation of gut microbiota and bile acid signaling [52]. Furthermore, it was illustrated that the anti-arthritis effect of kaempferol can be attributed to the rebalancing of gut flora and microbial metabolism [8].

**Figure 3:**
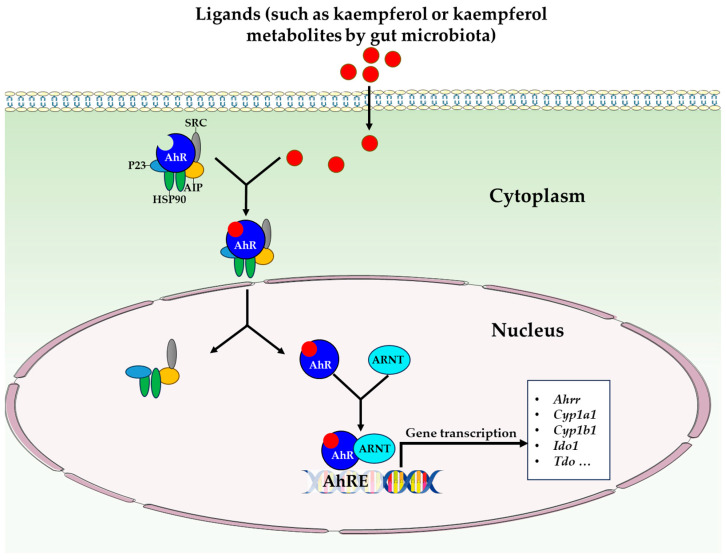
AhR signaling pathway. Abbreviations: AhR, aryl hydrocarbon receptor; AhRE, aryl hydrocarbon receptor responsive elements; Ahrr, AhR repressor; AIP, AhR-interacting protein; ARNT, aryl hydrocarbon receptor nuclear translocator; Cyp1a1, cytochrome P450 family 1 subfamily A member 1; Cyp1b1; cytochrome P450 family 1 subfamily B member 1; HSP90, heat shock protein 90; Ido1, indoleamine 2,3-dioxygenase 1; P23, co­chaperone P23; SRC, SRC protein kinase; Tdo, tryptophan 2,3-dioxygenase.

Most recently, the targeting of the aryl hydrocarbon receptor (AhR) with gut microbiota phenolic metabolites has been recognized as a crucial mechanism in the prevention and treatment of intestinal diseases [62,63]. Currently, dietary phenols (including kaempferol) have been extensively studied regarding their wide range of activities, which encompass antioxidant and anti-inflammatory properties. Nevertheless, it is necessary to consider their potential to modulate the AhR. The AhR is a ligand-dependent transcription factor that is a member of the basic helix-loop-helix (bHLH)-PAS family of transcription factors [64]. The AhR is extensively expressed in both immune and non-immune cells of the intestinal tract and its activation has been associated with the prognosis of intestinal conditions, including inflammatory bowel diseases [65]. Moreover, examinations of mice lacking AhR demonstrated numerous important beneficial functions associated with AhR activation [66]. In the absence of ligands, the AhR is located in the cytoplasm and forms a complex with molecular chaperones such as HSP90, p23, and XAP2 [67] (Figure 3). Upon ligand binding, the ligand–AhR complex enters the nucleus, resulting in the dissociation of the associated proteins. Subsequently, this complex binds to the ARNT (Aryl hydrocarbon receptor nuclear translocator), which in turn binds to the AHRE (Aryl hydrocarbon receptor responsive elements) located on the CYP1A1 promoter. As a consequence, the transcriptional activation of CYP1A1 and other phase II genes occurs [68]. According to reports, quercetin and kaempferol are natural and dietary ligands of the AhR, which exert varying effects on CYP1A1 transcription [69]. In addition, it was discovered that quercetin, resveratrol, and curcumin can act as indirect activators of the AhR [70]. Moreover, there is significant potential in gut phenolic metabolites for the prevention and/or treatment of intestinal inflammation. The question of whether intestinal kaempferol metabolites or kaempferol itself can regulate the AhR and its specific effects within the gut is an area that requires attention to address urgent clinical issues, such as inflammatory bowel diseases. Consequently, this topic is in its nascent stage of knowledge and should pique considerable interest within the scientific community due to its substantial impact on managing these diseases.

Additionally, despite the thorough validation of the efficacy of kaempferol in regulating intestinal physiological function and diseases via in vitro cell experiments and in vivo murine model experiments, no data are available regarding in vivo human trials. Furthermore, an extensive number of trials is required to investigate the precise dosage and duration of kaempferol in various pathological conditions. More importantly, there is a crucial need to examine the mechanism by which kaempferol regulates the specific pathologies of the intestine, with the aim of enhancing disease prevention and treatment, as well as obtaining a comprehensive understanding of specific intestinal diseases. Overall, future research endeavors should also prioritize investigating the specific dosage and duration of kaempferol in different pathological conditions, along with delving further into the underlying mechanisms of action that involve AhR regulation.

## 5. Conclusions

Kaempferol is a naturally occurring flavanol that is widely distributed across various plant genera as a secondary metabolite. Its chemical structure is characterized by phenyl rings and four hydroxyl substituents, making it an excellent radical scavenger. Kaempferol signifies a natural and potent antioxidant that possesses potential as a suitable functional agent for intestinal health. Numerous researchers have substantiated the idea that kaempferol holds significant potential for augmenting antioxidant capacity, mitigating gut inflammation, enhancing barrier function, and even aiding in the treatment of colorectal cancer and obesity. Based on current discoveries, the development and application of kaempferol emerges as a promising and evolving avenue for future research on intestinal health. Upcoming research endeavors should also prioritize investigating the precise dosage and duration of kaempferol administration in varying pathological conditions, while simultaneously delving deeper into the comprehensible mechanisms of action that involve AhR regulation.

## Figures and Tables

**Table 1:** Summary of kaempferol as a functional agent for intestinal health and disease (in vivo).

Subject	Stress Model	Kaempferol Dosage and Duration	Main Findings	Reference
C57BL/6J mice	Colitis induced by DSS	Dosage: 25, 50, 100 mg/kg/day (gavage) Duration: 30 days	Phenotype: ↓ disease activity index, colonic H&E score; Hemogram levels: ↑ RBC, HGB, ↓ WBC, LYM; Inflammation: ↓ colonic IL-1β, IL-6, TNF-α levels, colonic CRP, MPO activity, ↑ SOD activity; Barrier function: ↓ serum D-lactate, FITC-dextran levels; Colonic angiogenesis: ↓ VEGF-A, NO levels, ↑ IL-22BP protein level, ↓ VEGFR2, COX-2, TRAF6 protein levels, VEGFR2 (IHC score), MVD; Regulated gut microbiota.	[36]
C57BL/6J mice	Obesity induced by high-fat diet	Dosage: 0.1% (diet) Duration: 16 weeks	Phenotype: ↓ body weight, epididymal fat weight; Inflammation: ↓ macrophages cells, dendritic cells, MPO level, *F4/80*, *IL-6*, *TNF-α*, *MCP-1*, *IL-1β mRNA* levels in colon tissue; Intestinal barrier integrity: ↓ serum FITC-dextran level; ↑ *ZO-1*, *Occludin*, *Claudin-1* mRNA levels in colon tissue; TLR4/NF-κB pathway: ↓ TLR4, MyD88, cytosolic p65, nuclear p65 protein levels in colon tissue; Regulated gut microbiota.	[39]
C57BL/6J mice	Obesity induced by high-fat diet	Dosage: 200 mg/kg (diet) Duration: 8 weeks	Phenotype: ↓ body weight, iWAT, eWAT, pWAT; Regulated gut microbiota.	[59]
C57BL/6J mice	Colorectal cancer by impaired tumor suppress gene	Dosage: 50 mg/kg (gavage) Duration: 6 weeks	Phenotype: ↓ polyps count in intestinal tissue; Inflammation: ↓ *TNF-α* mRNA level in ileum; Signaling pathway: ↑ FXR mRNA and protein levels, ↓ β-Catenin protien level in intestinal tissues; Regulated gut microbiota and bile acid signaling.	[52]
C57BL/6 mice	Colitis induced by DSS	Dosage: 100 mg/kg (gavage) Duration: 20 days	Phenotype: ↑ colon lengh; Pathway: Regulation of BCRP and MRP2.	[35]
C57BL/6J mice	Colitis induced by DSS	Dosage: 50 mg/kg/day (gavage) Duration: 2 weeks	Phenotype: ↑ colon lengh; ↓ disease activity index, colon histological score; Inflammation: ↓ serum IL-1β, IL-6, TNF-α levels, colonic *IL-1β*, *IL-6*, *TNF-α*, *COX-2*, *MCP-1*, *iNOS*, *TLR4*, *NLRP3*, *MAPK1*, *NF-κB* mRNA levels, ↑ colonic *IL-10* mRNA level; Intestinal barrier integrity: ↓ serum FITC-dextran level; ↑ *ZO-1*, *Occludin*, *Claudin-1* mRNA levels and protein levels in colon tissue; LPS-TLR4-NF-κB pathway: ↓ serum LPS level; ↓ colonic protein levels of TLR4, MyD88, p-NF-κB-P65, and NLRP3; Regulated gut microbiota and serum metabolites.	[37]
ICR mice	Acute liver injury by alcohol	Dosage: 50, 100 mg/kg (oral administration) Duration: 2 weeks	Phenotype: ↓ liver histological scoring (interface hepatitis, lobular disarray, lobular inflammation, steatosis, and apoptosis); Histological scoring of ileum and colon: ↓ severity, degree of edema, location; Intestinal barrier integrity: ↑ Occludin, ZO-1 protein levels in ileum and colon; Butyrate receptor and transporter: ↑ SLC5A8, GPR109A protein levels in ileum and colon.	[46]
DBA/1J mice	Arthritis induced by collagen	Dosage: 200 mg/kg/day (gavage) Duration: 4 weeks	Phenotype: ↑ body weight, ↓ paw thickness, polyarthritis index, spleen index; Inflammation: ↓ plasma IL-1β, TNF-α, IL-6, IFN-γ, IgG levels; Regulated intestinal microbiota and microbial metabolism.	[8]
C57BL/6J mice	Colitis induced by DSS	Dosage: 0.3% (diet) Duration: 3 weeks	Phenotype: ↑ colonic length; ↓ disease activity index, colonic histological score, spleen weight; Inflammation: ↓ plasma PGE_2_, and LTB_4_ levels, colonic mucosa MPO level, colonic mucosa *COX-2*, *iNOS*, *TNF-α*, *IL-1β*, *IL-6 mRNA levels,* ↑ colonic mucosa *TFF3* mRNA level.	[38]

Abbreviations: ↑, increase; ↓, decrease; BCRP, breast cancer resistance protein; COX-2, cyclooxygenase-2; CRP, C-reaction protein; DSS, dextran sulfate sodium; eWAT, epididymal white adipose tissue; FXR, farnesoid X receptor; HGB, hemoglobin; IFN-γ, interferon-γ; IL-1β, interleukin-1β; IL-6, interleukin-6; IL-10, interleukin-10; IL-22BP, interleukin-22 binding protein; iNOS, inducible nitric oxide synthase; iWAT, inguinal white adipose tissue; LPS, lipopolysaccharide; LYM, lymphocyte; LTB_4_, leukotriene B_4_; MAPK1, mitogen-activated protein kinase 1; MCP-1, monocyte chemoattractant protein-1; MPO, myeloperoxidase; MRP2, multi drug resistanceassociated protein 2; MVD, microvascular density; MyD88, myeloid differentiation factor-88; NF-κB, nuclear factor kappa-B; NLRP3, nucleotide oligomerization domain (NOD)-like receptor 3; NO, nitric oxide; PGE_2_, prostaglandin E_2_; RBC, red blood cells; pWAT, perirenal white adipose tissue; SOD, superoxide dismutase; TFF3, trefoil factor family 3; TLR4, toll-like receptor 4; TNF-α, tumor necrosis factor-α; TRAF6, TNF receptor-associated factor 6; VEGF-A, vascular endothelial growth factor-A; VEGFR2, vascular endothelial growth factor receptor 2; WBC, white blood cell.

**Table 2:** The summary of kaempferol as a functional agent for intestinal health and disease (in vitro).

Subject	Stress Model	Kaempferol Dosage and Duration	Main Findings	References
Rat intestinal microvascular endothelial cells	Inflammation model induced by LPS and TNF-α	Dosage: 100 μmol/L Duration: 24 h	Inflammation: ↓ IL-6, ↑ IL-10 levels in culture supernatant; ↓ cell p-p65/p65 protein level, *IL-6*, *RELA/p65* mRNA levels; Angiogenesis: ↓ *VEGFR-2*, *VEGF* mRNA levels, VEGFR-2, p-Akt/Akt, VEGF-A, HIF-1α, p-p38/p38 protein levels; Tubular structure formation: ↓ tube nodes, total branching length; Barrier function: ↓ EB-Albumin permeability, Claudin-2 protein level, ↑ TEER, ZO-1, Occludin protein levels; Migration: ↓ cell mobility, p-p38, p-HSP27 protein levels.	[41]
IPEC-1 cells (intestinal porcine epithelial cells)	Oxidative stress model induced by diquat	Dosage: 10 μmol/L Duration: 24 h	Phenotype: ↑ cell viability, cell migration; Cell cycle arrest: ↓ G1 phase arrest, ↑ G2/M phase arrest, *Cyclin D1*, *CDK4*, *CDK6* mRNA levels, β-catenin protein level; Brrier function: ↓ FITC-dextran, ↑ TEER, ZO-1, ZO-2, Occludin, Claudin-4 protein levels; Oxidative damage: ↓ ROS production, ↑ T-Nrf2 protein level, *GSR*, *GSTA4*, *HO-1* mRNA levels, ↓ *GCLC* mRNA level; Apoptosis: ↓ cell apoptosis, mitochondrial depolarization, p-JNK/JNK protein level, ↑ Bcl-2/Bax, PARP-1 protein levels.	[32]
IEC-6 cells (Rat intestinal epithelial cells)	No stress treatment	Dosage: 5 μmol/L Duration: 24 h	Antibacterial ability: ↑ antibacterial activity, ↓ *E. coli* translocation; Barrier function: ↑ ZO-1, Occludin, Claudin-1 mRNA and protein levels; Pathway: ↓ RhoA, ROCK mRNA and protein levels.	[48]
IEC-6 cells	Cytotoxicity model induced by indomethacin	Dosage: 5 μmol/L Duration: 24 h	Cell cytotoxicity: ↑ cell viability, ↓ LDH release, ROS production, Ca^2+^ level; Barrier function: ↑ TEER, ↓ FD-4, ↑ ZO-1, Occludin, Claudin-1 mRNA levels and protein levels; Pathway: ↓ p-JNK/JNK, p-Src/Src protein levels.	[31]
Caco-2 cells (human colon epithelial cancer cells)	Deoxynivalenol-challenge model	Dosage: 100 μmol/L Duration: 24 h	Barrier function: ↑ TEER; Differentially expressed proteins: enriched in cell adhesion molecule, cell junction, cell junction assmbly; Pathway: regulation of PKA pathway and MAPK/ERK pathway.	[51]
Caco-2 cells cocultured with rat intestinal microvascular endothelial cells	Inflammation model induced by LPS	Dosage: 80 μmol/L Duration: 48 h	Inflammation: ↓ IL-8; Barrier function: ↑ TEER, ↓ FITC, ↑ ZO-1, Occludin, Claudin-2 protein levels; Pathway: ↓ p-p65/p65, p-I-κB/I-κB protein levels.	[49]
Rat intestinal microvascular endothelial cells	Inflammation model induced by LPS	Dosage: 50 μmol/L Duration: 3 h	Cytokine production: ↓ TNF-α, IL-1β, IL-6 levels in culture supernatant; Adhesion protein production: ↓ ICAM-1, VCAM-1 levels in culture supernatant; Pathways: ↓ TLR4, p-NF-κB p65/NF-κB p65, p-I-κB/I-κB, p-STAT/STAT.	[40]
Caco-2 cells	Deoxynivalenol-challenge model	Dosage: 100 μmol/L Duration: 24 h	Proliferation stage: ↓ ZO-1 protein level, ↑ FD-4 flux, Claudin-3 protein levels; Intermediate stage: ↑ TEER, Claudin-3 protein level, ↓ FD-4 flux, Claudin-4 protein level; Differentiated stage: ↑ TEER, Claudin-3 and ZO-1 protein level.	[50]
HT-29 cells (Human colon cancer cells)	Mutant *p53* gene	Dosage: 60 μmol/L Duration: 48 h	Cell apoptosis: ↑ early apoptotic cells; Caspase pathway: ↑ cleaved PARP, cleaved caspase-3, cleaved caspase-7, cleaved caspase-9 protein levels; Intrinsic apoptotic pathway: ↓ Bcl-xL, p-Akt, ↑ Bik, Bad (mitochondria) protein levels, ↓ Akt activity, ↑ mitochondrial membrane permeability, cytosol cytoochrome c protein level; Extrinsic apoptotic pathway: ↑ FAS-L, ↓ caspase-8, Bid protein levels, ↑ caspase-8 activity.	[56]
HT-29 cells	Mutant *p53* gene	Dosage: 60 μmol/L Duration: 24 h	Phenotype: ↓ Cell proliferation, ↑ cell cycle arrest.	[53]
Caco-2 cells	No stress treatment	Dosage: 100 μmol/L Duration: 48 h	Barrier function: ↑ TEER, ZO-2, Claudin-4 protein levels in whole cell lysate, ZO-1, ZO-2, Occludin, Claudin-1, Claudin-3, Claudin-4 protein levels in cell detergent-insoluble fraction.	[47]
Caco-2 cells	OVA-IgE complexes stimulation model	Dosage: 20 μmol/L Duration: 24 h	Inflammation: ↓ IL-8, MIP-3α levels; Pathway: ↓ p-ERK protein level.	[42]
HCT116 cells (Human colon cancer cells)	Mutant *p53* gene	Dosage: 60 μmol/L Duration: 24 h	Apoptosis: ↑ cell apoptosis; Pathway: ↑ cleaved PARP, cleaved caspase-3 protein levels.	[54]
SW480 cells (Human colon cancer cells)	No stress treatment	Dosage: 40 μmol/L (Co-treatment with TRAIL) Duration: 24 h	Apoptosis: ↑ cell apoptosis (Sub-G1); TRAIL receptor: ↑ DR4, DR5 protein levels.	[55]

Abbreviations: ↑, increase; ↓, decrease; Bax, Bcl-2-associated X protein; Bcl-2, B-cell lymphoma 2 protein; DR4, death receptor 4; DR5, death receptor 5; ERK, extracellular regulated protein kinases; GCLC, glutamate-cysteine ligase catalytic subunit; GSR, glutathione reductase; GSTA4, glutathione S-transferase 4; HIF-1α, hypoxia-inducible factor 1α; HO-1, heme oxygenase-1; HSP27, heat shock protein 27; ICAM-1, intercellular adhesion molecule-1; I-κB, inhibitor of κB; IL-1β, interleukin-1β; IL-6, interleukin-6; IL-8, interleukin-8; IL-10, interleukin-10; JNK, c-Jun N-terminal kinase; LDH, lactate dehydrogenase; MAPK, mitogen-activated protein kinase; MIP-3α, macrophage inflammatory protein-3 alpha; NF-κB, nuclear factor kappa-B; Nrf2, nuclear factor-E2-related factor 2; PARP, poly ADPribose polymerase; PKA, protein kinase A; RhoA, Ras homolog gene family member A; ROCK, Rho-associated protein kinase; ROS, reactive oxygen species; STAT, signal transducer and activator of transcription; TEER, transepithelial electrical resistance; TLR4, toll-like receptor 4; TNF-α, tumor necrosis factor-α; TRAIL, tumor necrosis factor-related apoptosis-inducing ligand; VCAM-1, vascular cell adhesion molecule-1; VEGF-A, vascular endothelial growth factor-A; VEGFR2, vascular endothelial growth factor receptor 2.

## Data Availability

Not applicable.
